# Sorafenib Combined with Chemoembolization for Locally Advanced Hepatocellular Carcinoma with Macroscopic Vascular Invasion: A Propensity Score Analysis

**DOI:** 10.3390/life11101066

**Published:** 2021-10-10

**Authors:** Gun Ha Kim, Sang Lim Choi, Jin Hyoung Kim, Ju Hyun Shim, Meshari Alali, Nayoung Kim

**Affiliations:** 1 Department of Radiology, Asan Medical Center, University of Ulsan College of Medicine, 88 Olympic-ro 43-gil, Songpa-gu, Seoul 05505, Korea; kimgh.rad@amc.seoul.kr (G.H.K.); radiolim@amc.seoul.kr (S.L.C.); me.alali@mu.edu.sa (M.A.); 2 Department of Gastroenterology, Asan Medical Center, University of Ulsan College of Medicine, 88 Olympic-ro 43-gil, Songpa-gu, Seoul 05505, Korea; s5854@amc.seoul.kr; 3 Department of Clinical Epidemiology and Biostatistics, Asan Medical Center, University of Ulsan College of Medicine, 88 Olympic-ro 43-gil, Songpa-gu, Seoul 05505, Korea; nyny0803@amc.seoul.kr

**Keywords:** hepatocellular carcinoma, macroscopic vascular invasion, transarterial chemoembolization, sorafenib

## Abstract

The purpose of this study was to compare the efficacy and safety of transarterial chemoembolization (TACE) plus sorafenib with those of TACE alone in patients with locally advanced hepatocellular carcinoma (HCC). Treatment-naïve patients with preserved hepatic reserve (Child–Pugh score ≤ 7) who received TACE plus sorafenib (*n* = 91) or TACE alone (*n* = 109) for locally advanced HCC with macrovascular invasion were retrospectively evaluated. Propensity score matching (PSM) was used to correct selection bias, and 63 pairs were created. In the entire study population, the median progression-free survival (PFS) and overall survival (OS) with TACE plus sorafenib were better than those with TACE alone. After PSM, the median PFS (7.0 vs. 4.3 months; *p* = 0.017) and OS (17.5 vs. 12.8 months; *p* = 0.049) were again significantly longer with TACE plus sorafenib than with TACE alone. Stratified Cox regression analysis and doubly robust estimation revealed that treatment type was significantly associated with both PFS and OS. In the subgroup analysis, TACE plus sorafenib did not show a significant survival benefit for patients with main portal vein or inferior vena cava invasion. Major complications were similar in both groups (*p* = 0.330). In conclusion, TACE plus sorafenib showed better survival outcomes than TACE alone in patients with locally advanced HCC.

## 1. Introduction

Hepatocellular carcinoma (HCC) is the second leading cause of cancer-related death worldwide, with about 800,000 deaths annually [1]. Unfortunately, a significant number of patients are diagnosed at an advanced stage, with Barcelona Clinic Liver Cancer (BCLC) stage C: cancer-related symptoms (performance status 1–2), Child–Pugh score A or B, macroscopic vascular invasion (MVI), and/or extrahepatic spread [2]. These patients have a poor prognosis with a median overall survival (OS) of approximately 6 months [3].

Sorafenib, a multi-kinase inhibitor with anti-proliferative and anti-angiogenic effects, has been the global standard of care for advanced HCC for a decade [3]. However, in two randomized trials, HCC patients with MVI showed poor response rates to sorafenib (only 2–3.3%) [4,5]. Therefore, in real-world practice, TACE is frequently considered as an alternative treatment for unresectable HCC with MVI [6]. Several studies showed that TACE could be safely performed and improved the survival outcomes in patients with MVI [7,8,9,10,11]. Moreover, TACE showed a direct therapeutic effect with lipiodol uptake against portal vein tumor thrombosis (PVTT) [12,13]. However, the efficacy of TACE alone for the treatment of HCC with MVI remains unsatisfactory [14,15].

Recently, many institutions have administered a combination treatment of TACE and sorafenib, as these two therapeutic options are expected to work synergistically. The acute hypoxia induced in surviving tumor cells by TACE may promote the upregulation of angiogenic growth factors, which can contribute to tumor recurrence or metastasis [16,17]. Sorafenib inhibits tumor cell proliferation by blocking angiogenic growth factors including vascular endothelial growth factor (VEGF) and platelet-derived growth factor (PDGF) [18]. However, several published studies show inconsistent results in intermediate or advanced HCC patients [19,20,21,22,23,24,25], and a few studies that compared TACE plus sorafenib with TACE alone for advanced HCC with MVI [26,27] suggest that TACE plus sorafenib shows significantly better OS outcomes than TACE alone, except for HCC patients with main PVTT [26,27]. However, these studies included significant numbers of patients with metastatic HCC (30–40%), whereas TACE is a local therapeutic option for liver-confined HCC and is not suitable for treating metastatic tumors. Thus, to evaluate the real OS benefit of TACE plus sorafenib in comparison with TACE alone, the comparison would best be limited to patients with locally advanced HCC (with MVI but without extrahepatic spread). Therefore, the purpose of our study was to compare the efficacy and safety of TACE plus sorafenib against that of TACE alone in patients with locally advanced HCC.

## 2. Materials and Methods

### 2.1. Study Patients

**Figure 1:**
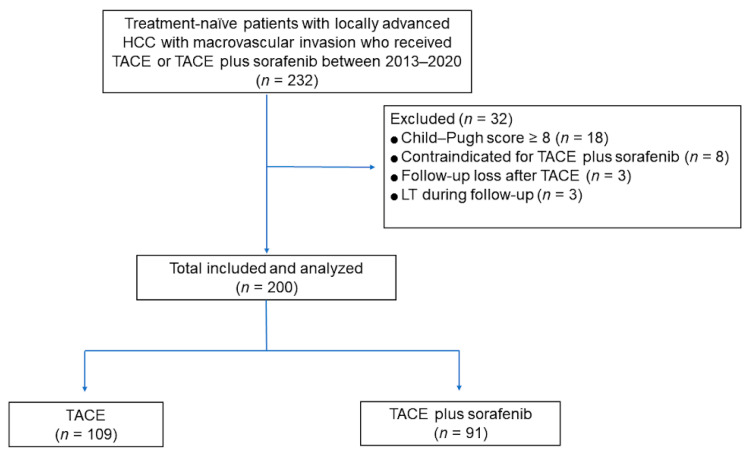
Flow diagram of the study population.

The study design was approved by our institutional review board, and the requirement for patient consent was waived because of the retrospective nature of the study. We reviewed 232 consecutive treatment-naïve patients with locally advanced HCC with MVI who received TACE plus sorafenib or TACE alone as first-line treatment between 2013 and 2020 at our institution. HCC was diagnosed according to the American Association for the Study of Liver Diseases (AASLD) or European Association for the Study of the Liver (EASL) criteria [2,28]. The presence and extent of MVI were evaluated using computed tomography (CT) or magnetic resonance imaging (MRI) [29]. The exclusion criteria were as follows: Child–Pugh score ≥ 8 (*n* = 18), any contraindication for sorafenib administration (*n*= 8; deterioration of liver function after TACE), follow-up loss after TACE (*n* = 3), and liver transplantation during follow-up (*n* = 3). Finally, a total of 200 patients were included (Figure 1). The physicians explained the 2 treatment options to all patients, and the final treatment decision was determined for each patient after considering the physician’s and patient’s preferences, as well as the cost.

### 2.2. Transcatheter Arterial Chemoembolization

The TACE procedure was performed as described previously [11,30], with a cisplatin dose of 2 mg/kg body weight. Using a microcatheter, an emulsion of cisplatin and iodized oil (3–20 mL; Lipiodol, Guerbet, Roissy, France) was infused into the target lobar, segmental, or subsegmental artery. This was followed by embolization with absorbable gelatin foam sponge (Gelfoam, Upjohn, Kalamazoo, MI, USA) slurry until near arterial flow stasis was achieved [11]. When a significant arterioportal or arteriovenous shunt was found during the procedure, embolization with Gelfoam was first performed to control the shunt to prevent a lipiodol shunt into the portal vein (PV) or a pulmonary embolism, after which the lipiodol/cisplatin emulsion was infused and Gelfoam embolization was finally carried out [31]. TACE was repeated every 4–6 weeks if residual viable HCC was observed on follow-up CT or MRI and the patient was without deterioration of liver function.

### 2.3. Sorafenib Therapy

Sorafenib treatment was performed as described previously [30]. Patients in the TACE plus sorafenib group started sorafenib at 400 mg twice daily on day 4 after the initial TACE. Then, sorafenib was administered on an interrupted schedule, with a 4–7 day interval before and after each subsequent TACE. If there was clinically significant toxicity of grade ≥ 2 (according to National Cancer Institute Common Terminology Criteria for Adverse Events, version 4.0), then the sorafenib doses were reduced, delayed, or briefly interrupted. Dose escalation or sorafenib rechallenge was determined when toxicity was diminished and the patient was able to tolerate the treatment well.

### 2.4. Definitions and Statistical Analyses

The radiologic response was assessed every 4–6 weeks with the use of dynamic CT or MRI, according to the modified Response Evaluation Criteria in Solid Tumors (mRECIST) guidelines [32]. The radiologic response was dichotomized into an objective response (including complete response [CR] and partial response [PR]) or non-regression (including stable disease [SD] and progressive disease [PD]) [33]. The best overall response during treatment was categorized as the final response [34]. The progression-free survival (PFS) period was defined as the time elapsed between treatment initiation and tumor progression (based on mRECIST) or death from any cause. The OS period was defined as the time from initial treatment to death from any cause. Major complications (grade 3 or 4 toxicity) were assessed and compared using the National Cancer Institute Common Terminology Criteria for Adverse Events, version 4.0.

Propensity score matching (PSM) was performed to minimize the effects of selection bias and potential confounders. The independent variables entered into the propensity model included age, sex, etiology, ECOG performance status, Child–Pugh score, maximal tumor size, tumor number, tumor type (nodular/infiltrative), tumor extent (uni/bilobar), presence of main (PV) or inferior vena cava (IVC) invasion, presence of liver cirrhosis, and levels of serum bilirubin, albumin, and alpha-fetoprotein (AFP). The model was then used to provide one-to-one matches between the two groups using the nearest-neighbor method [35].

The groups were compared before PSM using Student’s *t*-test for continuous variables and the chi-squared test for categorical variables. After PSM, the groups were compared using paired *t*-tests for continuous variables and the McNemar test or marginal homogeneity test for categorical variables. PFS and OS rates were calculated using the Kaplan–Meier method and compared using the log-rank test before PSM and the paired Prentice–Wilcoxon test after PSM. Multivariable Cox proportional hazards modeling with the backward elimination method was performed to identify independent factors showing associations with PFS and OS. Variables showing associations with a *p*-value < 0.05 and with marginal significance in the univariable analysis (*p*-value < 0.1) were entered into the multivariable model. After PSM, stratified Cox regression analysis and doubly robust estimation adjusted for factors significant in the univariable analysis were performed to identify the independent predictive function of the treatment type with regard to OS. Statistical analyses were performed using SPSS version 21 (SPSS, Inc., Chicago, IL, USA), and 2-sided *p*-values < 0.05 were considered statistically significant.

## 3. Results

### 3.1. Patients

During the study period, 200 patients received TACE plus sorafenib (*n* = 91) or TACE alone (*n* = 109) as first-line treatment for locally advanced HCC with MVI. The baseline characteristics of the patients are summarized in Table 1. Before PSM, both groups showed a predominance of males, and the TACE plus sorafenib group showed a younger mean age than the TACE-only group (56.2 ± 10.6 vs. 60.5 ± 11.1, respectively; *p* = 0.005). Chronic hepatitis B virus infection was the most prevalent etiology in both groups. The maximal tumor size, tumor type, ECOG performance status, Child–Pugh score, serum total bilirubin, albumin, and AFP levels did not differ significantly between the two groups, whereas the proportion of patients with a tumor number ≥ 4 was higher in the TACE-alone group than in the TACE plus sorafenib group (37% vs. 20%; *p* = 0.012), and the proportion of patients with main PV or IVC invasion was higher in the TACE plus sorafenib group than in the TACE-alone group (22% vs. 9%; *p* = 0.016). After PSM, the patients’ baseline characteristics were more balanced than they were before, and no statistically significant differences were observed between the two groups, including in age, tumor number, tumor involvement, and presence of main PV or IVC invasion.

### 3.2. Radiologic Response after Treatment

**Figure 2:**
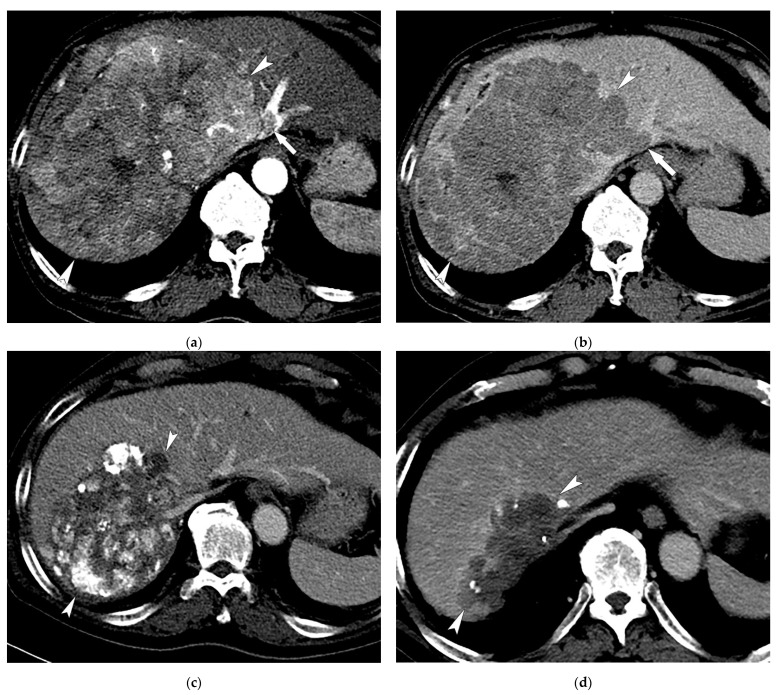
Images of a 59-year-old male patient with multinodular HCCs with MVI who received TACE combined with sorafenib treatment. Contrast-enhanced axial CT images in the arterial (**a**) and delayed (**b**) phases show a huge enhancing mass (17 cm in maximal diameter; arrowheads) invading the left hepatic vein (arrows). (**c**) CT image at 1 year after 4 sessions of TACE combined with sorafenib therapy shows necrotic change with lipiodol uptake in the tumor and a decrease in tumor size (13 cm; arrowheads). (**d**) CT image at 3 years after 6 sessions of TACE shows a further decrease in tumor size without tumor viability (9 cm; arrowheads).

During follow-up (median of 12.1 months; range, 0.5–99.3 months), 30 (33.0%) patients in the TACE plus sorafenib group showed a CR (Figure 2), 21 (23.1%) showed a PR, 34 (37.4%) had SD, and 6 (6.6%) had PD. In the TACE-alone group, 31 showed a CR (28.4%), 18 (16.5%) a PR, 40 (36.7%) had SD, and 20 (18.3%) had PD. The objective response (CR or PR) rates were 56.0% and 45.0% in the TACE plus sorafenib and TACE-alone groups, respectively (*p* = 0.156). After PSM, 24 (38.1%) patients in the TACE plus sorafenib group showed a CR, 15 (23.8%) showed a PR, 19 (30.2%) had SD, and 5 (7.9%) had PD. In the TACE-alone group, 20 showed a CR (31.7%) and 7 (11.1%) showed a PR, 24 (38.1%) had SD, and 12 (19.0%) had PD. The objective response rates were higher in the TACE plus sorafenib group than in the TACE-alone group, with borderline statistical significance (61.9% vs. 42.6%; *p* = 0.059).

### 3.3. Progression-Free Survival Analyses

**Figure 3:**
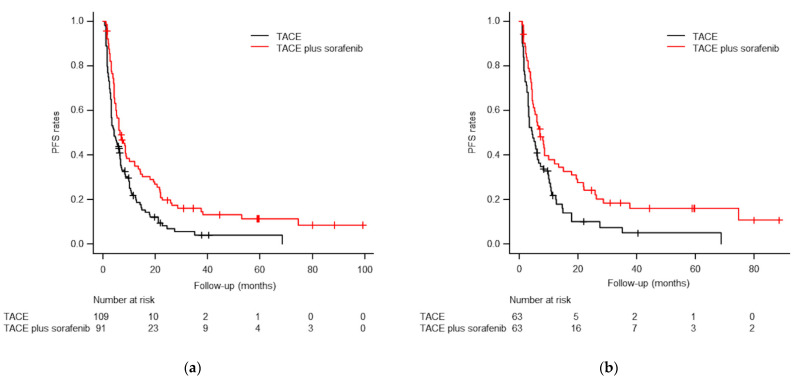
Kaplan–Meier curves of progression-free survival (PFS) rates before (**a**) and after (**b**) PSM. (**a**) The median PFS times were 6.5 months for TACE plus sorafenib and 4.2 months for TACE alone before PSM (*p* = 0.002). (**b**) The median PFS times were 7.0 months for TACE plus sorafenib and 4.3 months for TACE alone after PSM (*p* = 0.017).

During follow-up, 78 patients (85.7%) in the TACE plus sorafenib group and 99 (90.8%) in the TACE-alone group died or experienced HCC progression. The median PFS was significantly better in the TACE plus sorafenib group than in the TACE-alone group (6.5 vs. 4.2 months, respectively; *p* = 0.002; Figure 3). In the paired population after PSM, 51 patients (81.0%) in the TACE plus sorafenib group and 57 (90.5%) in the TACE-alone group died or experienced HCC progression. After PSM, the TACE plus sorafenib group showed longer median PFS than the TACE-alone group (7.0 vs. 4.3 months, respectively; *p* = 0.017; Figure 3).

### 3.4. Overall Survival Analyses

**Figure 4:**
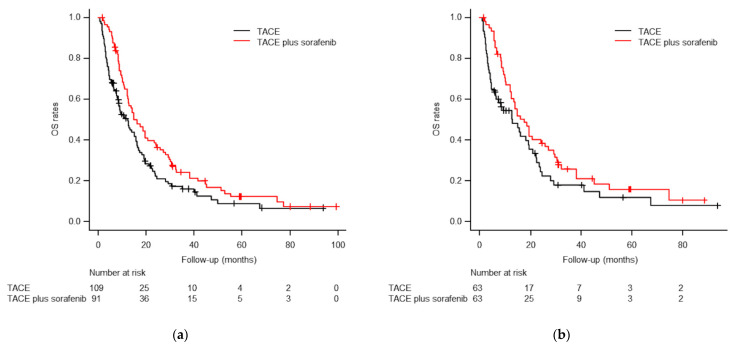
Kaplan–Meier curves of overall survival (OS) rates before (**a**) and after (**b**) PSM. (**a**) The median OS times were 16.0 months for TACE plus sorafenib and 12.2 months for TACE alone before PSM (*p* = 0.023). (**b**) The median OS times were 17.5 months for TACE plus sorafenib and 12.8 months for TACE alone after PSM (*p* = 0.049).

By the end of the follow-up period, 76 patients (83.5%) in the TACE plus sorafenib group and 87 (79.8%) in the TACE-alone group had died. The cumulative OS rates at 1, 3, and 5 years were 61.6%, 24.4%, and 12.4%, respectively, in the TACE plus sorafenib group, and 50.6%, 16.1%, and 9.1%, respectively, in the TACE-alone group. The median OS in the TACE plus sorafenib and TACE-alone groups was 16.0 months (95% confidence interval (CI), 12.2–20.6 months) and 12.2 months (95% CI, 8.4–15.7 months), respectively (*p* = 0.023; Figure 4). In the paired cohort after PSM, 49 patients (77.8%) in the TACE plus sorafenib group and 48 (76.2%) in the TACE-alone group died. The cumulative 1, 3, and 5 year OS rates in the paired cohort after PSM were 63.8%, 25.9%, and 15.9%, respectively, in the TACE plus sorafenib group, and 54.6%, 17.9%, and 11.9%, respectively, in the TACE-alone group. The TACE plus sorafenib group again showed better median OS than the TACE-alone group (17.5 vs 12.8 months; *p* = 0.049; Figure 4).

### 3.5. Multivariable Analyses

After the PSM, the stratified Cox regression and doubly robust estimation adjusted for factors significant in the univariable analysis revealed that treatment type was significantly associated with both PFS and OS (Table 2). The covariates adjusted for PFS were tumor number, tumor extent, bilirubin, and Child–Pugh score, whereas those adjusted for OS were maximal tumor size, tumor number, tumor extent, bilirubin, Child–Pugh score, and AFP.

### 3.6. Subgroup Analysis of OS Regarding Main PV or IVC Invasion

**Figure 5:**
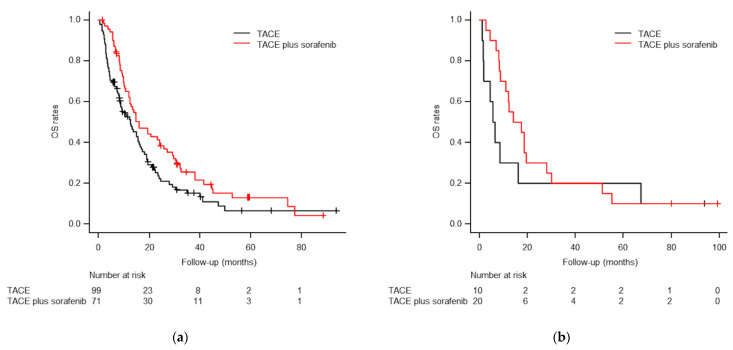
Subgroup Kaplan–Meier curves of overall survival (OS) rates in patients without (**a**) and with (**b**) main PV or IVC invasion. (**a**) The median OS times were 16.0 months for TACE plus sorafenib and 12.6 months for TACE alone in patients without main PV or IVC invasion (*p* = 0.030). (**b**) The median OS times were 14.0 months for TACE plus sorafenib and 5.6 months for TACE alone in patients with main PV or IVC invasion (*p* = 0.295).

Subgroup analysis showed that the median OS in patients without main PV or IVC invasion (*n* = 170) was significantly better in the TACE plus sorafenib group (16.0 months; 95% CI, 10.1–21.9 months) than in the TACE-alone group (12.6 months; 95% CI, 7.4–17.8 months; *p* = 0.030; Figure 5). In patients with main PV or IVC invasion (*n* = 30), the median OS was longer in the TACE plus sorafenib group (14.0 months; 95% CI, 2.4–25.6 months) than in the TACE-alone group (5.6 months; 95% CI, 2.7–8.5 months), but the difference was not statistically significant (*p* = 0.295; Figure 5).

### 3.7. Major Complications (Grade 3 or 4 Toxicities)

Major complications were observed in 14 (15.4%) patients in the TACE plus sorafenib group and 14 patients (12.8%) in the TACE-alone group (*p* = 0.330). The major complications in the TACE plus sorafenib group were grade 3 hepatic failure (*n* = 3), grade 3 hyperbilirubinemia (*n* = 2), grade 3 acute kidney injury (*n* = 2), grade 3 hand–foot skin reaction (*n* = 2), grade 3 liver abscess (*n* = 1), grade 3 peritonitis (*n* = 1), grade 3 thrombocytopenia (*n* = 1), grade 3 diarrhea (*n* = 1), and grade 4 hepatic failure (*n* = 1). The major complications in the TACE-alone group were grade 3 hepatic failure (*n* = 4), grade 3 liver abscess (*n* = 2), grade 3 peritonitis (*n* = 2), grade 3 fever (*n* = 1), grade 3 dyspnea (*n* = 1), grade 3 acute kidney injury (*n* = 1), grade 3 vasovagal syncope (*n* = 1), grade 4 acute renal failure (*n* = 1), and grade 4 acute respiratory distress syndrome (*n* = 1). After PSM, major complications were observed in 9 patients (14.3%) in the TACE plus sorafenib group and 8 patients (12.7%) in the TACE-alone group (*p* > 0.999).

## 4. Discussion

This study found that in patients treated for locally advanced HCC with MVI, a TACE plus sorafenib group had better survival outcomes than a TACE-alone group. After PSM, the median PFS and OS were significantly longer in the TACE plus sorafenib group than in the TACE-alone group. Stratified Cox regression analysis and doubly robust estimation also revealed that treatment type was significantly associated with both PFS and OS. The objective response rates were higher in the TACE plus sorafenib group than in the TACE-alone group, with borderline statistical significance. Similar rates of major complications were observed in both groups. Our study is different from others since previous studies [26,27] comparing TACE plus sorafenib with TACE alone for advanced HCC with MVI included significant numbers of patients with extrahepatic spread (30–40%). We excluded the patients with extrahepatic spread and decompensated liver function (Child–Pugh score ≥ 8) to evaluate the real OS benefit of TACE plus sorafenib in comparison with TACE alone, using a relatively large sample size with PSM. To the best of our knowledge, here we present the first study comparing TACE plus sorafenib with TACE alone for locally advanced HCC with MVI. A future randomized controlled trial (RCT) would be needed to confirm the efficacy of TACE plus sorafenib in study population with locally advanced HCC with MVI.

Previously, three RCTs on patients with unresectable HCC (mostly BCLC A and B patients) failed to show the benefits of combining TACE and sorafenib in comparison with TACE alone [19,20,36]. Recently, the TACTICS trial by Kudo et al. [21,37] compared the efficacy of TACE plus sorafenib with that of TACE alone in patients with unresectable HCC without MVI and extrahepatic spread (mostly BCLC A and B patients). In their study, the median OS was 36.2 months with TACE plus sorafenib and 30.8 months with TACE alone (HR, 0.861; 95% CI, 0.607–1.223; *p* = 0.40). Although TACE plus sorafenib did not show OS benefit when compared with TACE alone, the OS in the TACE plus sorafenib group in the TACTICS trial showed longer OS benefit (5.4 months) than was found in previous RCTs [19,20,36]. In addition, significantly better PFS was consistently observed in the TACE plus sorafenib group in their updated results (22.8 vs. 13.5 months; *p* = 0.02) [37]. The authors speculated that the major reason for the lack of a statistically significant OS benefit was the administration of a lot of post-trial treatments in the TACE-alone group (76.3%), which implies that OS may be limited as a study endpoint.

Park et al. [22] conducted an RCT to compare the efficacy of TACE plus sorafenib with sorafenib alone in patients with advanced HCC and did not find a significant OS benefit in the TACE plus sorafenib group compared with the sorafenib-alone group (12.8 vs. 10.8 months; *p* = 0.290), although the combination therapy did show significantly improved PFS (5.2 vs. 3.6 months; *p* = 0.009) and tumor response rate (60.6% vs. 47.3%; *p* = 0.005). However, for patients with first branch/main PV, hepatic vein, IVC, or atrial invasion, TACE plus sorafenib tended to show an OS benefit with borderline statistical significance (HR, 0.52; 95% CI, 0.27–1.02; *p* < 0.1). In the propensity score-matched cohort of the current study, the median PFS and OS (7.0 and 17.5 months, respectively) of the TACE plus sorafenib patients were longer than those reported by Park et al. [22], which may be due to the difference in the proportion of extrahepatic spread in the study cohorts (0% vs. 35.7%). Another RCT conducted by Yoon et al. [29] found that TACE plus radiotherapy showed better median PFS (7.5 vs. 2.8 months; *p* < 0.001) and OS (13.8 vs. 10.8 months; *p* = 0.04) than sorafenib alone in patients with locally advanced HCC with MVI. The median PFS and OS in the TACE plus sorafenib group in the current study are comparable with the results of Yoon et al. [29]. A very recent retrospective study using PSM [30] to compare TACE plus radiotherapy with TACE plus sorafenib in patients with advanced HCC with PVTT showed no significant difference in PFS and OS between the two groups.

In previous studies [8,9,10,38], TACE alone showed survival benefits compared with best supportive care in patients with main PVTT. However, combination of sorafenib with TACE was not able to identify additional survival benefit with no significant differences between the two treatment groups (*p* = 0.06 [26] and *p* = 0.588 [27]). Our study also showed no statistically significant difference in OS between treatment groups in cases with main PV or IVC invasion (*p* = 0.295). However, the median OS values were 14.0 months for TACE plus sorafenib and 5.6 months for TACE alone, and the statistical significance may have been low because of type II error, likely caused by the small sample sizes (*n* = 20 vs. *n* = 10, respectively).

According to the GIDEON study [39], there were global variations in the combination of TACE with sorafenib in patients with HCC. Longer duration of sorafenib treatment was associated with improved survival outcomes [19,21,36], however, there is no evidence-based consensus on when to administer sorafenib—before or after the first TACE session. There are studies in which sorafenib was administered not only before the first TACE session [19,20,21,22,24], but also after the first TACE session [26,27,30,40]. Thus, future comparative studies are needed to determine the optimal timing of this combination treatment and to develop clinical guidelines.

Recently, a phase 3 trial (IMbrave150) [41] showed superior PFS and OS in an atezolizumab plus bevacizumab group than in those treated with sorafenib as the first-line treatment for unresectable HCC (including 81% BCLC C patients). A change in the standard systemic therapy for advanced HCC to atezolizumab plus bevacizumab could influence the interpretation of previous trials. However, the efficacy of atezolizumab plus bevacizumab for advanced HCC with MVI remains unclear, and a future comparative study is therefore required to determine the better option in HCC patients with MVI including main PV or IVC invasion: atezolizumab plus bevacizumab or TACE combined with systemic treatment (sorafenib, lenvatinib, or atezolizumab plus bevacizumab). In addition, diverse treatment modalities for advanced HCC are evolving, and several RCTs including combination treatments with immunotherapy are ongoing [42,43].

The limitations of this study include its non-randomized retrospective nature, which may make it vulnerable to selection bias. However, any potential bias was minimized by performing PSM. Second, this is a single-center study, which may limit the generalizability of the results. Therefore, our results may not be applicable to patients with locally advanced HCC in other countries because of differences in patient demographics and the underlying etiologies of liver disease. Third, other subsequent treatments due to disease progression or unacceptable major complications might have affected OS. However, we also analyzed PFS to compare the efficacy of TACE plus sorafenib with TACE alone.

In conclusion, TACE plus sorafenib showed better survival outcomes (both PFS and OS) than TACE alone in patients with locally advanced HCC with MVI. However, the OS benefit of TACE plus sorafenib compared to TACE alone in the treatment of HCC with main PV or IVC invasion remains unclear. Future study is needed to find a better regimen for a TACE-based combination with new drugs.

## Figures and Tables

**Table 1:** Patient characteristics before and after propensity score matching.

	Study Population before PSM			Study Population after PSM		
	TACE + Sorafenib	TACE	*p*-Value	TACE + Sorafenib	TACE	*p*-Value
Patients	91	109		63	63	
Age (mean ± SD)	56.2 ± 10.6	60.5 ± 11.1	0.005	58.0 ± 10.0	58.1 ± 10.4	0.941
Male sex, *n* (%)	78 (86)	87 (80)	0.351	52 (83)	51 (81)	>0.999
Etiology			0.819			0.866
HBV	71 (78)	81 (74)		48 (76)	48 (76)	
HCV	6 (7)	9 (8)		3 (5)	4 (6)	
Others	14 (15)	19 (18)		12 (19)	11 (17)	
ECOG PS			0.225			0.838
0	57 (63)	78 (72)		44 (70)	42 (67)	
1	34 (37)	31 (28)		19 (30)	21 (33)	
Maximal tumor size (cm, mean ± SD)	9.4 ± 3.5	9.0 ± 4.5	0.412	9.1 ± 3.4	9.1 ± 4.4	0.996
Tumor number ≥ 4, *n* (%)	18 (20)	40 (37)	0.012	15 (24)	14 (22)	>0.999
Tumor extent			0.054			>0.999
Unilobar	70 (77)	69 (63)		47 (75)	46 (73)	
Bilobar	21 (23)	40 (37)		16 (25)	17 (27)	
Bilirubin (mg/dL, mean ± SD)	0.85 ± 0.47	0.84 ± 0.40	0.866	0.86 ± 0.49	0.86 ± 0.40	0.953
Albumin ≤ 3.5 mg/dL, *n* (%)	44 (48)	56 (51)	0.777	31 (49)	34 (54)	0.735
AFP ≥ 400 ng/mL, *n* (%)	51 (57)	64 (59)	0.775	37 (59)	36 (57)	>0.999
Child-Pugh score B, *n* (%)	7 (8)	17 (16)	0.135	6 (10)	6 (10)	>0.999
Tumor type			0.257			0.265
Nodular	36 (40)	52 (48)		26 (41)	19 (30)	
Infiltrative	55 (60)	57 (52)		37 (59)	44 (70)	
Main PV or IVC invasion			0.016			0.773
Yes	20 (22)	10 (9)		7 (11)	9 (14)	
No	71 (78)	99 (91)		56 (89)	54 (86)	

SD, standard deviation; HBV, hepatitis B virus; HCV, hepatitis C virus; AFP, alpha-fetoprotein; ECOG PS, Eastern Cooperative Oncology Group performance status; PV, portal vein; IVC, inferior vena cava; TACE, transcatheter arterial chemoembolization; PSM, propensity score matching.

**Table 2:** Cox proportional hazards analyses of TACE plus sorafenib versus TACE alone on PFS and OS.

Analysis	HR	95% CI		*p*-Value
**PFS**				
Unadjusted	0.622	0.459	0.842	0.002
Adjusted	0.651	0.476	0.890	0.007
Propensity score matched *	0.463	0.310	0.693	<0.001
Propensity score matched and adjusted for selected variables †	0.443	0.286	0.687	<0.001
**OS**				
Unadjusted	0.701	0.514	0.955	0.024
Adjusted	0.750	0.543	1.035	0.080
Propensity score matched *	0.583	0.399	0.853	0.005
Propensity score matched and adjusted for selected variables ‡	0.574	0.377	0.872	0.009

HR, hazard ratio; CI, confidence interval; PFS, progression-free survival; OS, overall survival. * Cox proportional hazard models, with robust standard errors that accounted for the clustering of matched pairs. † Adjusted for tumor number, tumor extent, bilirubin, and Child–Pugh score, which were significant in the univariable analysis. ‡ Adjusted for maximal tumor size, tumor number, tumor extent, bilirubin, Child–Pugh score, and AFP, which were significant in the univariable analysis.

## Data Availability

Not applicable.
